# Incidence Rate of Incisional Hernia Post Liver and Kidney Transplant at a Tertiary Center in Riyadh, Saudi Arabia

**DOI:** 10.7759/cureus.20223

**Published:** 2021-12-07

**Authors:** Abdulrahman M Alhassan, Mohammed N Alghunaim, Ayyob A Alqarni, Abdulkareem M Abdullah, Mohammed K Altoyan, Abdullah S Alharbi, Faisal A Alhusain

**Affiliations:** 1 Medicine, King Abdulaziz Medical City, National Guard Health Affairs, Riyadh, SAU; 2 General Surgery, King Abdulaziz Medical City, National Guard Health Affairs, Riyadh, SAU; 3 Medicine, College of Medicine, King Saud bin Abdulaziz University for Health Sciences, Riyadh, SAU; 4 Emergency Medicine, King Abdulaziz Medical City, National Guard Health Affairs, Riyadh, SAU

**Keywords:** post transplant incisional hernia, factors of incisional hernia, incisional hernia, liver transplant, kidney transplant

## Abstract

Background

Incisional hernia post organ transplant increases morbidity and impacts quality of life among patients undergoing abdominal organ transplants.

Objectives

To estimate the incidence rate of incisional hernia and the factors associated with incisional hernia among patients who underwent liver and kidney transplants.

Methods

This was a retrospective cohort study in which all patients from 2015 to 2020 who underwent liver and/or kidney transplants and met inclusion criteria were involved.

Results

A total of 424 patients who received transplantation surgery were included. Out of them, 287 patients (67.6%) underwent kidney transplants while 132 patients (31.1%) underwent a liver transplant. Additionally, five patients (1.1%) received both liver and kidney transplantation. Fourteen patients (3.3%) experienced incisional hernia across all samples. A higher incidence rate was noticed among patients with liver transplants compared to kidney transplants (6.81% in the liver group vs 1.7% in the kidney group), which showed a statistical significance between the two groups (P-value= 0.007). In multivariate analysis, surgical site infection (SSI), donor type, acute organ rejection, mycophenolate mofetil (MMF), and diabetes were all not predictors of incisional hernia among the patients.

Conclusion

Incisional hernia incidence in between the groups was within the global range of incisional hernia incidence among abdominal organ transplant patients, with a higher incidence among liver transplant patients. All factors associated with incisional hernia, such as SSI, DM, and old age, didn’t show significance as predictors to incisional hernia formation among the samples.

## Introduction

Hernias in the abdominal wall are one of the common findings that can affect all age groups. It is known as an abnormal projection of the peritoneal sac through the abdominal wall. Surgeries in the abdomen are considered as one of the causes. Most of the hernias are asymptomatic which can be discovered during checkups; however, patients with hernia can present with various symptoms such as swelling and a heavy feeling in the abdomen [1]. In incisional hernia repair, a synthetic mesh is used that can be done as an open surgery or a laparoscopic surgery [2].

Organ transplant is a surgical procedure aimed to transplant an organ from a living or deceased donor to a living recipient in need that meets qualification criteria. This procedure has been lifesaving to many patients with end-stage organ failure. Historically, the first successful organ transplant was done in the mid-1950s [3,4], and since that time, transplantation has become a crucial medical option to treat patients with organ failure. Moreover, in the United States (US), 95 organ transplants take place each day, and more than 15,000 organ transplants are done in the US yearly [5]. In Saudi Arabia, 1980 liver transplants had been done from 1990 to 2016 [6]. Furthermore, 779 renal transplants were performed in more than 12 centers in 2015 in Saudi Arabia [7]. The records have also shown that more than 60 transplants of the pancreas have been done since 1990, with 18 transplants done in 2017 alone [7,8]. The increased number of patients needing organ transplants as the main treatment has created a demand for more centers for transplants. Therefore, the number of kidney transplant centers, for instance, in Saudi Arabia has increased from 12 to 16 in 2017 [8].

Incisional hernia post abdominal organ transplant is a possibility like in any other abdominal surgeries. The incidence of incisional hernia post liver transplant has been estimated as ranging up to 43% in some studies [9], even though low rates have been documented [10,11]. On the other hand, the incidence rate of incisional hernia after kidney transplant has reached up to 7% [12]. Furthermore, the incidence of incisional hernia post-pancreatic transplant in the first year was estimated to be 2.5% [11]. Despite the different lifetime risk and incidence rates, multiple factors have shown to be playing a role in the formation of incisional hernia, some of which are technical related factors and patient related factors. Some of the patient related risk factors are smoking, malnutrition, obesity, and connective tissue disorders. Whereas, technical related factors are increased wound tension, failing of surgical material, and poor surgical technique [13]. Additionally, surgical site infection (SSI), gender, age, type of donor, and utilization of immunosuppression agents have been linked as factors to incisional hernia post-organ transplant [12,14,15].

This study aimed to assess the incidence rate of incisional hernia and its risk factors among patients who underwent kidney or liver transplantation at a tertiary hospital in Riyadh, Saudi Arabia.

## Materials and methods

This is a retrospective cohort study through chart review of all the patients who underwent abdominal organ transplantation (liver, kidney, or both) at King Abdulaziz Medical City, National Guard Health Affairs, Riyadh, Saudi Arabia from 2015 to 2020. The charts were reviewed by the research team to confirm the transplantation surgery. Then, the relevant data was collected and analyzed from electronic records. The data included gender, age, and type of organ transplant as grouping variables. On the other hand, incidence rate of incisional hernia and risk factors were considered as outcome variables. The study was approved by the King Abdullah International Medical Research Center IRB (RC20/494/R).

Study subjects

The study involved all patients from 2015 to 2020 with a history of abdominal organ transplant with the following inclusion criteria First, the age of the patients was ≥ 18 years. Second, we only included patients who underwent liver and/or kidney transplantations in adult patients (18 years old and above) in King Abdulaziz Medical City, National Guard Health Affairs, Riyadh, Saudi Arabia from 2015 to 2020. All patients that didn’t meet the inclusion criteria or passed away within one month after transplantation were excluded.

Data collection and variables

The method used in this study for data collection was as the following. First, the patients who underwent solid organ transplantation (liver, kidney, or both) were reviewed to confirm the surgery was performed. Once the surgery was confirmed, and the inclusion criteria met, the relevant data was collected. The research members collected demographic data, formation of incisional hernia at site of transplantation, the type of immunosuppressive medications used for induction and maintenance, presence of diabetes, model end-organ liver disease (MELD) score for liver recipients, presence of wound infection, type of donor, history of abdominal surgery before the transplant surgery, diagnosed with acute organ rejection, and development of delayed graft function for kidney recipients, which was defined as the need for dialysis within the first seven days after transplant.

Statistical analysis

IBM SPSS Statistics for Windows, Version 22.0 (Released 2013, IBM Corp., Armonk, New York) was used in the analysis. Categorical variables were expressed in the form of the number and percentage, and their particular groups were compared using the chi-square test. Numerical data were reported using mean and standard deviation (SD). A logistic regression model was run to assess the predictors of incisional hernia. All studied variables have been used individually in univariate logistic regression except variables that are characteristical to one group of patients, such as delayed graft function for kidney transplants. Then, the same variables were used again in multivariate logistic regression to predict incisional hernia. The incidence rate is reported as a percentage with 95% CI. A p-value < 0.05 is considered a statistical significance between the groups.

## Results

**Figure 1:**
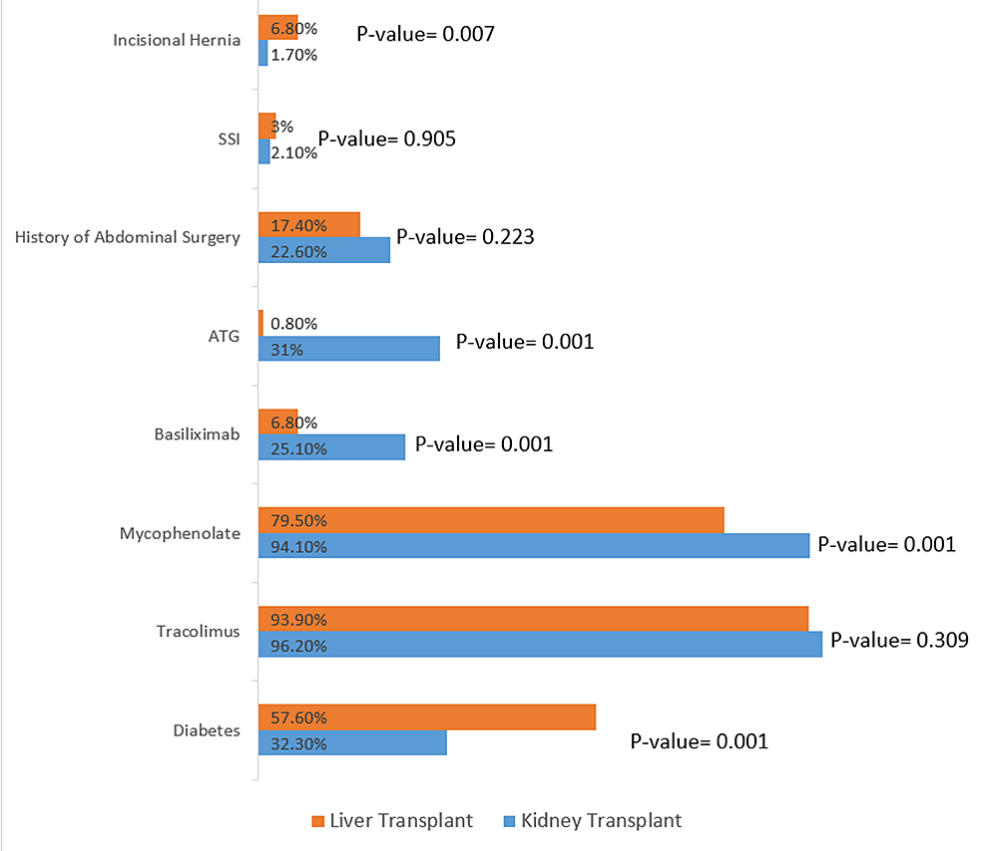
Different variables with the corresponding p-value in relation to the type of transplant ATG: Anti-thymocyte globulin; SSI: Surgical site infection

A total of 424 patients who received transplantation surgery were included. Out of them, 287 patients (67.6%) received kidney transplants, and 132 patients (31.1%) received liver transplants (Table 1). Moreover, five patients (1.1%) received both liver and kidney transplantation, and their characteristics are presented in Table 2. In the kidney transplant group, 168 (58.5%) patients were male and 85 (64.4%) liver transplant patients were male (p-value=0.255). The age category has shown statistical significant in between the groups (p-value= 0.001), with the mean age of patients with kidney transplant (45.5±15.3) lower than liver transplant patients (58.7±10.3) (Table 1). In the liver transplant group, a higher proportion of patients, 76 (57.5%), has had diabetes in comparison to the diabetics portion in the kidney transplant, 91 (32.3%), which showed to be statistically significant (p-value<0.001). In regard to the medications, there was significant association noticed in patients treated with mycophenolate, anti-thymocyte globulin (ATG), and basiliximab to type of transplant (p-value <0.001) (Table 1, Figure 1). Majority of patients among the groups have had BMI less than 30. However, there was no statistically significant difference between the studied groups regarding BMI (p=0.73) (Table.1). Most patients in the liver transplant group have received transplants from deceased donors, 82 (62.1%). In contrast, live donors were the majority among kidney transplant patients, 223 (77.7%), which is statistically significant (p-value= 0.001). The mean pre-transplantation MELD score for liver transplant was 19.0±6.9, and delayed graft function was encountered in 21 (7.4%) kidney transplant patients. Out of the included patients, 65 (22.6%) patients had a history of previous abdominal surgery in the kidney transplant group in contrast to 23 (17.4%) patients in the liver transplant group (p=0.22) (Table 1, Figure 1).

**Table 1:** Description of demographic data of liver and kidney transplants ATG: Anti-thymocyte globulin; MELD: Model end-organ liver disease; SSI: Surgical site infection

Variables	Type of transplant		
	Kidney	Liver	p-value
	Number (%) Mean±SD	Number (%) Mean±SD	
Number of patients	287	132	
Gender			
Male	168 (58.5%)	85 (64.4%)	0.25
Female	119 (41.5%)	47 (35.6%)	
Age	45.57±15.30	58.79±10.35	
<60 years	212 (73.9%)	55 (41.7%)	0.001
≥60 years	75 (26.1%)	77 (58.3%)	
BMI			
<30	152 (54.9%)	72 (56.7%)	0.73
≥30	125 (45.1%)	55 (43.3%)	
Diabetes	91 (32.3%)	76 (57.6%)	0.001
Tracolimus	276 (96.2%)	124 (93.9%)	0.30
Mycophenolate	270 (94.1%)	105 (79.5%)	0.001
Basiliximab	72 (25.1%)	9 (6.8%)	0.001
ATG	89 (31.0%)	1 (0.8%)	0.001
History of abdominal surgery	65 (22.6%)	23 (17.4%)	0.22
Delayed graft function kKidney only)	21 (7.4%)		___
MELD score (liver only)		19.02±6.96	___
Donor			
Living	223 (77.7%)	50 (37.9%)	0.001
Deceased	64 (22.3%)	82 (62.1%)	
Acute organ rejection	31 (11.0%)	11 (8.3%)	0.41
SSI	6 (2.1%)	3 (2.3%)	0.90
Incisional hernia	5 (1.7%)	9 (6.8%)	0.007

**Table 2:** Basic characteristic of patients with both liver and kidney transplants ATG: Anti-thymocyte globulin; DGF: Delayed graft function; SSI: Surgical site infection

Patients have Both Liver and Kidney transplants	Variables	Number of patients. Mean±SD
	Number of patients	5
	Gender (Female)	4
	Age (≥ 60 years)	2
	BMI (≥30)	2
	Diabetes	2
	Tracolimus	5
	Mycophenolate	5
	Basiliximab	1
	ATG	1
	History of abdominal surgery	0
	DGF	1
	Donor (deceased)	5
	Acute organ rejection	1
	SSI	0
	Incisional hernia	0

The total number of patients who experienced post-transplant incisional hernia was 14 (3.3%) (Table 1). The incidence was relatively higher in patients who received liver transplants, 6.81% (N=9), in contrast to patients who received renal transplants, 1.7% (N=5), which showed a statistical significance (p= 0.007) (Figure 1). No patient has encountered incisional hernia in the kidney and liver transplant groups.

Table 3 and Table 4 represent the predictors of incisional hernia. In multivariate analysis, there was no clinical significance noticed in gender (OR=1.66, 95%CI 0.25-3.28, p-value=0.88), history of abdominal surgery (OR=0.65, 95%CI 0.37-4.73, p-value=0.66), and donor type (OR=0.38, 95%CI 0.51-6.99, p-value=0.33). Furthermore, there was no significance encountered with diabetes (OR=2.75, 95%CI 0.17-2.38, p-value=0.50) and SSI (OR=3.81, 95%CI 0.05-7.96, p-value=0.74) as predictors for incisional hernia (Table 4). Age lower than 60 years has been shown to be at lower risk to incisional hernia in univariate analysis (OR=0.14, 95%CI 0.04-0.53, p-value=0.003). Moreover, in comparison to liver transplants, kidney transplants appeared to be protective to incisional hernia in univariate analysis (OR=0.24, 95%CI 0.08-0.73, p-value=0.013) (Table 3). However, there was no statistical significance of age (OR=0.14, 95%CI 0.04-0.53, p-value=0.052) and type of transplant (OR=0.24, 95%CI 0.08-0.73, p-value=0.43) in multivariate logistic regression model. Medications have shown to be negative to predict incisional hernia in both univariate and multivariate models (Table 3 and Table 4).

**Table 3:** Univariate logistic regression of the variables studied as predictors to incisional hernia ATG: Anti-thymocyte globulin; SSI: Surgical site infection

Variables	OR	95% CI		P-value
		Lower	Upper	
Gender (male)	1.66	0.51	5.40	0.39
Type of transplant (kidney transplant)	0.24	0.08	0.73	0.013
Age (< 60 years)	0.14	0.04	0.53	0.003
BMI (< 30)	1.46	0.48	4.45	0.50
Diabetes	2.75	0.90	8.37	0.07
Tracolimus	3.80	0.78	18.35	0.09
Mycophenolate	0.64	0.08	5.07	0.67
Basiliximab	1.45	0.31	6.62	0.62
ATG	1.66	0.36	7.58	0.50
History of abdominal surgery	0.65	0.20	2.13	0.48
Donor (living)	0.38	0.13	1.13	0.08
Acute organ rejection	1.50	0.32	6.96	0.60
SSI	3.81	0.44	32.80	0.22

**Table 4:** Multivariate logistic regression model for patients' characteristics, medications, and transplant related outcomes as predictors to incisional hernia ATG: Anti-thymocyte globulin; SSI: Surgical site infection

Variables	OR	95% CI		P-value
		Lower	Upper	
Gender (male)	1.66	0.51	5.40	0.88
Type of transplant (kidney transplant)	0.24	0.08	0.73	0.43
Age (< 60 years)	0.14	0.04	0.53	0.052
BMI (< 30)	1.46	0.48	4.45	0.24
Diabetes	2.75	0.90	8.37	0.50
Tracolimus	3.80	0.78	18.35	0.06
Mycophenolate	0.64	0.08	5.07	0.23
Basiliximab	1.45	0.31	6.62	0.57
ATG	1.66	0.36	7.58	0.96
History of abdominal surgery	0.65	0.20	2.13	0.66
Donor (living)	0.38	0.13	1.13	0.33
Acute organ rejection	1.50	0.32	6.96	0.77
SSI	3.81	0.44	32.80	0.74

## Discussion

This study aimed to find the incidence rate of incisional hernia among patients post organ transplantation with the factors associated with it. We have found a 1.7% incidence of incisional hernia in kidney transplant patients and 6.8% in liver transplant patients, with the total incidence of incisional hernia among all the patients being 3.34%. A similar study done in 2015 showed a higher incidence rate with the total incidence of incisional hernia being 7.5% [11]. We have noticed a higher rate of incisional hernia in liver transplant patients compared to kidney transplant patients with statistical significance (p-value= 0.007) (Figure.1). Likewise, multiple studies assessed the incidence rate among kidney transplant recipients, and the reported incidence rate of incisional hernia was ranging from 1.1-7% [12,16,17], whereas the reported incidence rate after liver transplant was from 1.1-43% [9,15,18,19]. According to Garmpis et al. [14], type of incisions such as the bilateral subcostal incision and the Mercedes incision could play a role in incisional hernia formation among liver transplant recipients. On the scope of that, both subcostal and Mercedes incisions are large incisions, which may be a factor contributing to the incisional hernia incidence among liver transplant patients [20]. The incidence rate that has been shown among liver transplants in this study was noticed to be similar to the local study done by Hegab et al. [21].

Multiple factors may contribute to incisional hernia formation. We have found that type of transplant and age < 60 were predictors of incisional hernia only on univariate logistic regression (Table 3). However, in the multivariate analysis, we couldn’t identify evidence supporting the transplant type and age as factors for incisional hernia formation (Table 4). SSI, older age, and BMI were reported as factors influencing incisional hernia [11,22,23]. Our study, however, didn’t identify significance of these factors to incisional hernia. Factors such as male sex, abdominal re-intervention, and living donor were linked to incisional hernia among patients post liver transplant [10,14]. On the other hand, female gender, history of smoking, obesity, delayed graft function, increased age, and deceased donor were considered as factors for incisional hernia in kidney transplant patients [11,12,24]. Additionally, immunosuppressive medications were reported as factors in incisional hernia among organ transplant patients [20]. The absence of mycophenolate mofetil (MMF) has been reported as a risk associated with incisional hernia [11,12]. Alternatively, MMF was linked to incisional hernia in liver transplant patients [25]. MMF was associated with incisional hernia in kidney transplant patients when compared to azathioprine [26]. In this study, there was no association of incisional hernia to immunosuppression therapy that has been studied, with the emphasis that only multivariate analysis was used and included both groups. 

Surgical technique and site have been associated with incisional hernia. Midline incision, Mercedes incision, increase tension on fascia, left iliac fossa implantation of kidney, parietal musculofascial closure in one single layer, and wound length are counted as risk factors of incisional hernia [14,19,23,24]. The history of abdominal surgery wasn’t significant to be a factor in incisional hernia formation in this study. Our findings were consistent with large-scale studies aimed to find the incidence and risk of incisional hernia [23]. SSI is a major risk in developing incisional hernia among transplant and non-transplant patients. We were surprised that the total incidence of SSI in our study was 2.1% (N=9 patients), compared to the higher rate of SSI of more than 5% among different transplant sites and types [27]. The low number of patients in the study having SSI could be related to our center’s capacity to minimize the patients’ pre and perioperative risks such as DM, donor screening, maximizing intra-operative status of the patient, and providing adequate coverage of antimicrobial, antiviral, and antifungal regiments. The incidence of SSI in the two groups was similar (2.1% and 2.3%, respectively).

The study has encountered statistical and non-statistical limitations. Due to the very small number of patients having both kidney and liver transplants, we couldn’t define it as a group to analyze further in the regression model. The low number of patients having incisional hernias contributed to limitation in studying the two groups individually. Therefore, MELD score and delayed graft function couldn’t be assessed as risk factors. Surgical-related data such as surgical technique, surgical site, wound size, time interoperation, and post-surgery complication haven’t been assessed in this study.

## Conclusions

The incidence rate of incisional hernia post liver transplant was higher when compared to kidney transplant, and there was no incidence reported in the group who underwent both kidney and liver transplant. The incidence seems to be consistent with other studies, and surgical related factors could play a role in incisional hernia incidence among patients post transplant. However, there were no relation found in this study between gender, age, type of transplant, BMI, DM, immunosuppression medications, and history of abdominal surgeries to incisional hernia. Low incidence of SSI was found in the samples, which can be justified by optimal antimicrobial coverage and maximizing the health status of the patients. Finally, clinical trial studies are needed to help in assessing the best surgical technique and interventions to minimize incisional hernia rates.
